# Screening of wheat genotypes for the presence of common bunt resistance genes

**DOI:** 10.1016/j.sjbs.2021.02.013

**Published:** 2021-02-16

**Authors:** Aigul Madenova, Zagipa Sapakhova, Serik Bakirov, Kanat Galymbek, Gulzira Yernazarova, Alma Kokhmetova, Zhenis Keishilov

**Affiliations:** a Al-Farabi Kazakh National University, 050040, Al-Farabi Avenue 71, Almaty, Kazakhstan; b Institute of Plant Biology and Biotechnology, 050040, Timiryazev Street 45, Almaty, Kazakhstan; c Abai Kazakh National Pedagogical University, 050010, Dostyk Avenue 13, Almaty, Kazakhstan; d Kazakh National Agrarian Research University, 050010, Abai Avenue 8, Almaty, Kazakhstan

**Keywords:** Wheat, Genotypes, Common bunt, Resistance genes, *Bt* genes, Molecular markers

## Abstract

Common bunt is known to cause grain yield and quality losses in wheat due to bunt ball formation and infestation of the grain. The aim of this study is to identify for sources of resistance to common bunt in wheat genotypes using phytopathological and molecular methods. In general, studied 60 Kazakh and foreign wheat genotypes were found 15 samples with the *Bt9*, *Bt8* and *Bt11* genes. Carriers of the *Bt10* gene include the five varieties. The four resistance genes, *Bt8, Bt10, Bt11, Bt9*, and *Bt10* were identified in the Karasai variety. Phytopathological and molecular screening of Kazakh and foreign wheat genotypes selected 18 with genes for resistance to the disease. According to evaluation on an artificial infection 19 varieties showed an immune type of reaction. These varieties will be used in breeding programs as donors to create resistant varieties against the common bunt. Thus, approaches can reduce the level of fungicides use and the most effective method to control the common bunt.

## 1 Introduction

Wheat common bunt is one of the most significant biotic barriers to wheat production worldwide. To implement the tasks of reducing the spread of the disease, obtaining an uninfected agricultural product, the treatment of seeds with fungicides is widely used. The use of modern drugs pesticides the direct losses of the agricultural product; they effectively destroy spores on the seeds and in the soil. However, the method of using seed dressing protectants damages the environment and human health. This method of protection is not economically viable and unacceptable in organic farming (Yorgancılar, 2016). With the introduction of effective fungicides around 50 years ago, most wheat breeding programs shifted their priorities away from the selection for bunt resistance. An increase in organic farming over the last two decades gave rise to a renewed interest in bunt diseases of wheat (Matanguihan et al. 2011). Wheat is a staple food in many countries around the world. It is the main product for 35% of the population and comprises 20% of world consumption. Fungal disease during epiphytotic years can cause maximum yield losses. Fungal diseases include common bunt diseases, rust diseases as well as leaf spot diseases, etc. (Goates, 2012, Galymbek et al., 2017, Kokhmetova et al., 2016, Kokhmetova et al., 2019, Madenova et al., 2019a, Madenova et al., 2020 Morgounov et al., 2015). Common bunt, caused by fungi from the genus *Tilletia*, is widespread around the world where wheat is grown; it is a serious problem for the production of this crop. This disease is one of the most harmful for grain crops; it destroys grains, turning them into a black dense mass of spores. When the seed planted without any chemical treatment for a few years, this damage could reach 75–90% (Akan et al., 2014). Seed treatments with fungicides could be used as an effective tool to manage common bunt. However, genetic resistance is a better option for reducing exposure to chemical seed treatments and could be applied in organic systems (Ciucã, 2011, Matanguihan, 2011). No specific mycotoxins have been identified until now but the high levels of trimethylamine contained in common bunt sori cause the typical smell of rotten fish in diseased crops (Matanguihan et al., 2011, Chen et al., 2016). Common bunt has been associated with wheat production since ancient times and common bunt infection has been ever-present (Gaudet and Menzies 2012). Common bunt is widespread in all regions of Central Asia, including southern and southeastern Kazakhstan, where winter wheat is mainly cultivated. In the countries of North Africa and Central Asia, common bunt is ranked second after rust in terms of harmfulness; it infects 5–7% of the crop. In these countries, only 40% of the seeds are chemically treated. In the 1990 s, in the southern, southeastern and eastern regions of Kazakhstan, sowing of unsealed seeds led to a sharp increase in the prevalence of winter wheat durum and dwarf bunt. In 1997–1998, in separate farms of southern and southeastern Kazakhstan, harvested winter wheat grain was unsuitable for processing into flour and animal feed. Common and dwarf bunt affected 15–38% of the wheat ears, i.e. one caused the loss of up to one-third of the crop (Koishybayev 2018).

According to Koishybayev and Muminjanov (2016), in the early 2000 s, the area of distribution and the degree of damage to winter wheat crops by common bunt in Kazakhstan increased. For many years, phytopathological research has been conducted in Kazakhstan and abroad to study wheat collection around the world and identify sources of resistance to common bunt. According to the literature, there has been a fairly large set of sources of resistance to common bunt diseases identified in various ecological and geographical areas of the world. Thus, Koishybayev and Muminjanov (2016) established an artificial infectious background. More than 200 commercials and promising varieties of winter wheat in Central Asia and the Caucasus region are highly resistant to common bunt: Pamyat 47, Eritrosperium 350 (Kazakhstan), Meyanopus 223, Eritrosperium 9945, Keremet, Azibrosh (Kyrgyzstan), Sanzar-4, Kokhraba, Tamara (Uzbekistan), Atay-85, Sham, Dzhager (Tadzhikistan), Ugur, Karabakh and Leukurum 1207 (Azerbaidzhan). Relative to the population of *Tilletia caries* from Kazakhstan and Tajikistan, isogenic wheat lines with *Bt5, Bt8, Bt9*, and *Bt10* genes have high efficiency.

Currently, the most common method for controlling seed infection is chemical control. There is a direct correlation between embryonic infection and the appearance of a common bunt in the field. Fungicidal seed treatment does not decrease the severity of this disease; the solution to the issue of infection of common bunt crops is still relevant. According to many researchers, the fight against common bunt should be complex and include chemical, agrotechnical and biological methods, as well as growing of resistant varieties.

Resistance against the common bunt disease is expressed with the Bt genes. The Bt genes are determined by using the race differential set (Matanguihan et al., 2011). Sixteen resistance genes, designation Bt1-Bt15 and Btp, have been identified (Goates, 2012) and gene bank accessions (Goates and Bockelman, 2012). The *Bt10* is genetically mapped to the terminal end of chromosome 6DS (Menzies et al. 2006, Singh et al., 2016) and *Bt9* mapped as a distinct factor on the distal end of chromosome 6DL (Steffan et al. 2017).

One way to increase the effectiveness of breeding programs is to use molecular markers in addition to classical methods. The use of molecular markers will increase the ability to assess the resistance of genes to plant diseases and pests. Al-Maaroof et al. (2016) showed that the *Bt2, Bt4, Bt7, Bt10, Bt13, Bt14*, and *Bt15* genes were overcome in the conditions of Iraq, while the *Bt3, Bt5, Bt6, Bt9, Bt11* and *Bt12* genes under an artificial infectious background effectively contained infection.

There are practically no common bunt resistant wheat varieties in production. The creation of sustainable wheat varieties ensures the stability of production, especially during epiphytotic years, and also ensures quality, cost and provides sanitary and epidemiological safety in the field. Given the increasing cost of seed protectants and their environmental insecurity, the most affordable way to protect plants while reducing the pesticide load on agrocenoses is to cultivate varieties that are resistant to common bunt. The objective of our study is to identify resistant genotypes to common bunt, among the genetic diversity of winter wheat samples against the background of artificial infection, for southeastern Kazakhstan.

## 2 Materials and methods

### 2.1 Experimental site and plant materials

The research was carried out at the quarantine nursery of the Kazakh Research Institute of Agriculture and Crop Production, which located in the Almalybak village, Almaty region, Republic of Kazakhstan (43°13′09″ N. 76°41′17″ E) during the three growing seasons 2017/2018, 2018/2019, 2019/2020. The experimental material comprised of 61 bread wheat (*Triticum aestivum* L.) varieties from the Kazakh and foreign breeding program, including 53 varieties – Kazakh, 1-Russian, 2- Uzbek, 2-Ukrainian, 1-Kyrgyz, 1-Tajik, and 1-Turkey wheat varieties (Table 2). The Yakar-99 and Bogarnaya 56 are used as a susceptible control for common bunt. The experiment was laid out using complete block design with three replications. The size of each plot was 1 m^2^. Evaluation of infected head by common bunt was carried out by counting of spikes of 50 plants.

### 2.2 Inoculum and inoculation

Evaluation of an artificial infectious background allows one to determine the degree of infectability of the studied wheat samples, cull susceptible samples, and purposefully conduct work. Common bunt spores (*T. caries*) collected in the Almaty region of Kazakhstan served as the epidemic material. Many years of research have confirmed the prospects for the method of dry seed clogging according to 1 g of spores per 100 g of seeds. This procedure successfully balances the ratio of the pathogen and seeds, and thus there is a very high degree of plant damage with an extremely simple spore-based technique. To speed up the process, the spore mass is not weighed repeatedly; rather, it is selected with a special measure and strictly dosed based on a certain mass of chlamydospores (Koishybayev and Muminjanov, 2016) The optimum temperature for *T. caries* and *T. laevis* spore germination and subsequent seedling infection is 10–13 °C. Given these pathogen features, winter wheat should be sown at a later date, namely early for spring wheat. For inoculation spores from 2017 to 2017/2018, 2018 to 2018/2019 and 2019 to 2019/2020, which were stored at 4 °C we used. We counted sick and healthy ears and calculated the percentage of damage. In all three growing seasons, 90–100% of bunt infections were observed in the susceptible control cultivar of Bogornaya 56 and Yakar-99. Such an outcome indicated the success of inoculation. We conducted all field management and agricultural practices according to the Ministry of Agriculture recommendations. We calculated the infection percent for each genotype from the first two experiments at the dough stage by counting the number of healthy and infected spikes per each meter, according to Dodov and Todorova (1974). The modified method utilised the following parameters: R = resistant (infection percent 0–5%); I = intermediate resistant (infection percent 6–25%); S = susceptible (infection percent 26–50%); and HS = highly susceptible (infection percent 51–100%). We averaged data from each differential line to determine virulent resistant (0–10% spike infection) or virulent susceptible (11–100% spike infection), in accordance with Hoffmann and Metzger (1976).

### 2.3 DNA extraction

The polymerase chain reaction (PCR) used to determine carriers of resistance genes. The genomic DNA from 5-day-old wheat seedlings was extracted using the CTAB method (Riede and Anderson, 1996). The carriers of *Bt* genes were identified on the basis of PCR using the developed protocols. STS markerswereused to identify *Bt*-gene carriers. The PCR total volume was 25 µl, with 2.5 µl of 10x buffer for Taq polymerase, 2.5 µl of dNTPs (2.5 mm of each nucleotide), 0.5 µl of each primer, 0.5 µl of Taq polymerase, and 18 µl of Milli-Q H20. Amplified DNA fragments were separatedusing electrophoresis in a 2% agarose or 8% polyacrylamide gel in a TBE buffer (45 mmM Tris-borate, 1 mM ethylenediaminetetraacetic acid (EDTA), pH 8; (Chen, Line and Leung, 1998). BioRad amplifier (TM100 Thermal cycler, Singapore) was used with the following thermal cycling conditions: initial denaturation at 94 °C for 5 min; 45 cycles of 1 min at 94 °C, 1 min at 45 °C and 2 min at 72 °C; and a final elongation for 7 min at 72 °C. The PCR programs were modified depending on the identified gene.

### 2.4 Statistical analysis

The Prism program (GraphPad 5.0) was used for statistical analyses, such as One-way ANOVA and *t*-test to compare the means of each year as well as each parameter.

## 3 Results

Molecular markers associated with bunt resistance genes could aid the development of resistant cultivars by facilitating screening for resistance and the introgression of bunt resistance genes in wheat cultivars with good agronomic genotypes (Ciucã, 2011). For instance, marker-assisted selection has become increasingly popular for wheat breeders in Canada in the last 15 years. Breeding for disease resistance, including common bunt, plays an important role in the application of marker-assisted selection (Randhawa et al. 2013).

An effective way to combat common bunt is to create disease-resistant varieties. Therefore, one of the main tasks of selection is the continuous search for sources of Bt genes, which confer resistance to common bunt and subsequently implement them in wheat varieties. As a result of molecular analysis, we identified carriers of *Bt* genes in 60 Kazakh and foreign wheat samples.

In connection with the need to develop organic selection methods, it is necessary to create new varieties that will be resistant to such a formidable disease as common bunt without the use of chemical treatments of seeds. However, there are currently no wheat varieties for this target. In this regard, the use of molecular markers can help find sources of resistance to this disease. Because molecular markers in organic farming accelerate the production of resistant samples for a common bunt. The objective of the project under investigation is the introduction of marker technology Marker Assisted Selection to create new varieties of wheat with effective *Bt* genes for subsequent implementation in farms focused on organic farming.

The *Bt9* gene is located at the distal end of chromosome 6D. The *Bt10* gene is also based on this chromosome. Their possible grip or co-location was suggested. After the comparison, it was found that the *Bt9* and *Bt10* genes are two distinct genes of wheat resistance to common bunt, located, respectively, at the 6DL and 6DS ends of chromosome 6D (Rasmussen, 2016, Steffan et al., 2017).

**Fig. 1:**
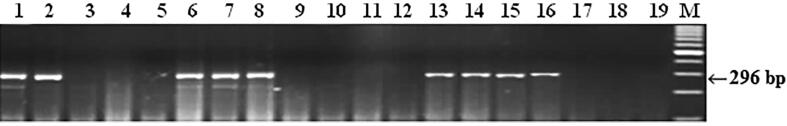
DNA amplification products of winter wheat samples using primers to the Xgpw 7433 locus linked to the gene Bt9.

To classify *Bt9* gene carriers, PCR amplification was performed using SSR (Table 1) pmarkers such as 7433 markers (Steffan et al. 2017). As a positive control for the identification of *Bt* gene carriers, The *Bt-9* M82-2098 isogenic line was used, which the *Bt9* resistance gene was previously identified. The size of DNA fragment for *Bt9* gene carriers is 296 base pairs (bp). This fragment was identified in several varieties: Dinara, Yegemen 20, Zhalyn, Kazakstanskaya 75, Karasai, Mereke75, Matai, Naz, Sultan 2, Sultan 95, Sanzar 8, Steklovidnaya 24, Raminal, Farabi and Yubileynaya 75 (Fig. 1). In general, out of screened 60 Kazakh and foreign wheat vatieties 15 varieties identified as a sources of the *Bt9* gene (Table 2).

**Table 1:** Selection of molecular markers linked to genes for resistance to smut.

Genes	Localization	Type of marker	Marker name	Sequence	T°	The fragment size (bp)	Reference
*Bt9*	6D	SSR	Xgpw7433	GTACATGGAAAGAGACCAACA CCA CGCTGAGCAAGGACGATAG	60 °C	296	Steffan et al. 2017
*Bt10*	6DS	SCAR	FSD RSA	GTT TTATCTTTTTATTTC CTCCTCCCCCCA	44 °C	275	Laroche et al., 2000
*Bt8 Bt10 Bt11*	3B	*SSR*	Xgwm114	ACAAACAGAAAATCAAAA CCCG ATCCATCGCCATTGGAGTG	58 °C	180 160 120	Goates and Mercier (2009)

**Table 2:** Molecular screening of Kazakh and foreign wheat varieties and disease severity under an artificial infection condition, (Almaty region, Almalybak, 2018–2020).

Variety	Origin*	Bt genes	Infection, Type^**^	Severity, %			
				Average	2017/2018	2018/2019	2019/2020
Diana	KZ	none	MS	25.00	24.00	29.00	22.00
Dinara	KZ	Bt9, Bt8, Bt10	R	0.00	0.00	0.00	0.00
Yegemen	KZ	none	S	53.00	52.00	49.00	58.00
Yegemen 20	KZ	Bt9, Bt8, Bt10	R	2.00	0.00	0.00	6.00
Zhalyn	KZ	Bt9	MR	10.33	0.00	10.00	21.00
Zhetisu	KZ	Bt8, Bt11	MR	13.00	4.00	13.00	22.00
Zhadyra	KZ	none	MS	27.67	16.00	26.00	41.00
Kazakstanskaya 16	KZ	none	MR	24.00	9.00	18.00	45.00
Kazakstanskaya 75	KZ	Bt9, Bt8, Bt11	MR	10.00	4.00	10.00	16.00
Krasnovodapadskaya	KZ	Bt8	MR	19.67	5.00	21.00	33.00
Krasnovodapadskaya 25	KZ	none	R	1.00	1.00	0.00	2.00
Krasnovodapadskaya 210	KZ	none	MS	29.67	7.00	24.00	58.00
Karabalykskaya winter	KZ	none	MR	12.00	8.00	10.00	18.00
Karabalykskaya Ostistaya	KZ	Bt10	MR	8.67	4.00	8.00	14.00
Karabalykskaya 101	KZ	none	R	1.67	3.00	0.00	2.00
Konditerskaya	KZ	none	MS	28.00	26.00	26.00	32.00
Karasai	KZ	Bt8, Bt9, Bt10, Bt11	R	1.33	4.00	0.00	0.00
Karaspan	KZ	none	MR	5.33	2.00	6.00	8.00
Karlygash	KZ	none	MR	18.33	14.00	16.00	25.00
Kokbiday	KZ	Bt10	MR	12.00	4.00	12.00	20.00
Keremet	KZ	none	MR	8.00	6.00	6.00	12.00
Koksu	KZ	none	R	1.00	3.00	0.00	0.00
Kyzylbiday	KZ	none	R	0.00	0.00	0.00	0.00
Knyazhna	KZ	none	MR	20.33	20.00	13.00	28.00
Intensivnaya	KG	none	MS	37.67	11.00	36.00	66.00
Manshuk	KZ	none	MR	9.33	6.00	0.00	22.00
Myra	KZ	none	MR	12.00	11.00	6.00	19.00
Mereke 70	KZ	none	MS	39.33	53.00	35.00	30.00
Mereke 75	KZ	Bt9, Bt11	R	2.67	8.00	0.00	0.00
Matai	KZ	Bt9	R	0.00	0.00	0.00	0.00
Mironovskaya 808	UK	none	MS	44.67	39.00	43.00	52.00
Naz	KZ	Bt9, Bt8, Bt11	R	2.67	3.00	0.00	5.00
Nureke	KZ	Bt8	MR	11.00	2.00	12.00	19.00
Odesskaya 120	UK	none	MR	13.67	9.00	13.00	19.00
Progress	KZ	none	MR	15.00	0.00	16.00	29.00
Prezident	KZ	none	S	56.00	51.00	68.00	49.00
Pyrotrix 50	KZ	none	MS	30.00	16.00	34.00	40.00
Taza	KZ	none	MS	25.67	0.00	26.00	51.00
Tungysh	KZ	none	MR	17.33	7.00	15.00	30.00
Talimi-80	KZ	none	R	0.00	0.00	0.00	0.00
Sultan 2	KZ	Bt9, Bt8	R	0.00	0.00	0.00	0.00
Sultan 95	KZ	Bt9, Bt11	R	0.67	2.00	0.00	0.00
Sanzar 8	UZ	Bt9	R	0.33	1.00	0.00	0.00
Sapaly	KZ	Bt8, Bt11	R	0.00	0.00	0.00	0.00
Steklovidnaya 24	KZ	Bt9	R	0.00	0.00	0.00	0.00
Umanka	KZ	none	R	4.33	2.00	0.00	11.00
Ulugbek 600	UZ	none	MS	31.00	28.00	31.00	34.00
Ramin	KZ	none	MR	15.00	10.00	8.00	27.00
Raminal	KZ	Bt9	R	1.33	0.00	0.00	4.00
Rasad	KZ	none	R	4.33	2.00	0.00	11.00
Reke	KZ	none	MS	42.67	28.00	32.00	68.00
Farabi	KZ	Bt9	MR	15.67	10.00	13.00	24.00
Sharora	TJ	none	R	4.00	8.00	0.00	4.00
Yuzhnaya 12	KZ	none	R	1.33	0.00	0.00	4.00
Yubileynaya 60	KZ	none	S	51.33	35.00	52.00	67.00
Yubileynaya 75	KZ	Bt9	MR	6.67	2.00	6.00	12.00
Almaly	KZ	Bt10	MR	24.67	0.00	25.00	49.00
Bezostaya 1	RU	none	R	4.67	6.00	5.00	3.00
Bayandy	KZ	none	MR	23.00	20.00	22.00	27.00
Bogornaya 56	КZ	none	S	67.33	97.00	68.00	37.00
Yakar-99	TR	none	S	97.00	94.00	98.00	99.00
Average	15.79	11.38^**^	14.38^**^	21.60^****^			
*t*-test^**^	6.25^****^	5.61^****^	3.89^****^				

*Origin: KZ = Kazakhstan; KG = Kyrgyzstan; UZ = Uzbekistan; RU = Russian Federation; UK = Ukraine; TR = Turkey; TJ = Tajikistan;

**Infection type: R = resistant (infection percent 0–5%); I = intermediate resistant (infection percent 5–25%); S = susceptible (infection percent 26–50%); HS = highly susceptible (infection percent 51–100%); classification according to Dodov and Todorova (1974) and Krivchenko (1984).

***The results are significant at p-value *< 0.05; **< 0.01. ***< 0.001. ****< 0.00001.

**Fig. 2:**
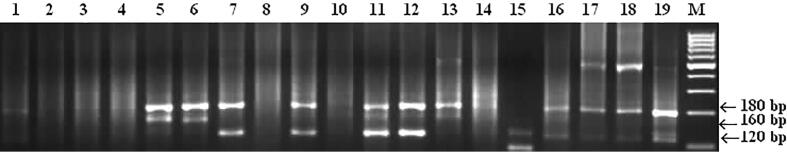
DNA amplification products of winter wheat samples using primers to the Xgwm114 locus linked to the genes Bt8, Bt10, Bt11.

To identify carriers of the *Bt8, Bt10*, and *Bt11* genes PCR was performed (Fig. 2) using the Xgwm114 markers (Goates et al. 2012). Xgwm114 primers amplified fragments of 180, 160, and 120 bp linked to the *Bt8, Bt10,* and *Bt11* genes. The Turkish genotype PI178383 (McIntosh, 2013), which identified *Bt8, Bt9*, and *Bt10* genes and one unknown resistance gene were used as a positive control for identification of these gene carriers. The *Bt8* gene was detected in seven varieties such as Dinara, Yegemen 20, Zhetisu, Sapaly, Krasnivodapadskaya, Kazakstanskaya 75, Nureke. The *Bt10* gene was identified in two varieties (Dinara and Yegemen), the *Bt11* gene in four varieties (Zhetisu, Sapaly, Kazakstanskaya 75, Naz) (Fig. 2). Among 60 Kazakh and foreign studied varieties, the *Bt8* gene was detected in the following 10 varieties Dinara, Yegemen 20, Zhetisu, Sapaly, Krasnivodapadskaya, Kazakstanskaya 75, Nureke, Sultan 2 and Sapaly. The *Bt10* gene was found in 3 varieties, the *Bt11* gene in 7 samples, and as well as two *Bt8* and *Bt11* genes in 5 varieties. The Karasai winter wheat variety was found as a combination of four following resistance genes: *Bt8, Bt11, Bt9*, and *Bt10,* (Table 1).

**Fig. 3:**
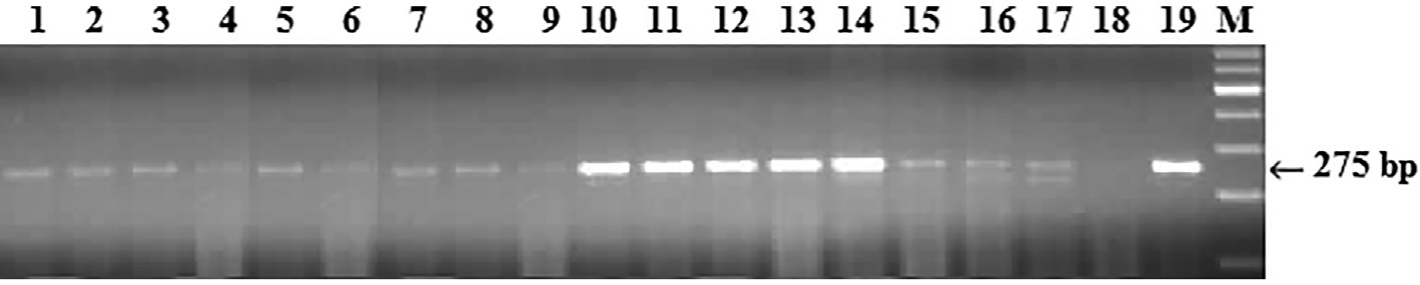
DNA amplification products of winter wheat samples using primers to the FSD/RSA locus linked to the genes Bt10.

Among the common bunt resistance genes, much attention has been paid to *Bt10* because, according to the literature, this gene is the most effective against gene to common bunt around the world. To search for the *Bt10* gene was used primers for the SCAR marker FSD/RSA (Laroche et al., 2000). The FSD/RSA marker is typical for *Bt10* gene carriers, which amplified 275 bp DNA fragment. The *Bt10* gene selected in the following varieties: included Karabalykskaya ostistaya, Karasai, Kokbiday and Almaly (Table 2). Overall, molecular screening of 60 Kazakh and foreign wheat varieties showed that four carriers of the *Bt10* gene were identified (Fig. 3).

Data of the adult plant reaction revealed a range of response levels of the tested wheat varieties to common bunt during all seasons. Among 60 wheat varieties tested, twenty-nine varieties demonstrated resistance to common bunt in the first growing season (2017/2018), and twenty-three varieties offered resistance in the second growing season (2018–2019), while only eighteen varieties showed resistance in the third growing season (2019/2020). Among 60 Kazakh and foreign wheat varieties tested only seven varieties exhibited resistance response for three years (2017/2018, 2018/2019, 2019/2020), which showed a high resistant reaction (0%). These varieties include Dinara, Kyzylbiday, Matai, Talimi-80, Sultan 2, Sapaly and Steklovidnaya 24. Fifteen varieties presented reaction from 0.33% to 4.67%, which classified as resistant varieties to common bunt infection. There were twenty two moderate susceptible varieties (infection percent 5–25%) to disease. The group of moderate susceptible varieties (26–50% infected ears) was five varieties (Konditerskaya, Intensivnaya, Mereke 70, Mironovskaya 808, Pyrotrix 50, Taza, Ulugbek 600, Reka) in 2017/2018 and eleven varieties (Diana, Yegemen, in 2018/2019, as well as fifteen varieties in 2019/2020. Five varieties of wheat (Yegemen, President, Yubileynaya 60, Bogornaya 56, Yakar-99) were highly susceptible and affected by the disease (Table 2). The rest varieties exhibited susceptible response recording infected ears more than 50%.

The mean values of productive traits such as grain number (GN), grain weight (GW), 1000-kernel weight (TKW) and Normalized difference vegetation index (NDVI), as well as rankings of wheat varieties are shown in Table 3. There were twelve resistant varieties showed the highest level of GN, GW, TKW and NDVI (Yegemen 20, Karasai, Kyzylbidai, Mereke 75, Matai, Naz, Sultan 2, Sapaly, Steklovidnaya 24, Rasad, Sharora, Bezostaya 1). The most of high productive varieties is commercial wheat varieties, which commonly grown in Kazakhstan. The second growing season (2018/2019) was the most productive year compared two growing seasons (2017/2018 and 2019/2020). However, in the first season disease severity was less than other two seasons.

**Table 3:** Mean values (2018–2020), varieties and rankings for productivity traits.

Variety	GN		GW		TKW		NDVI	
	pcs	rank	g	rank	g	rank	unit	rank
Diana	41.18	58	1.47	60	35.63	57	0.70	45
Dinara	48.28	46	2.15	40	41.12	39	0.67	58
Yegemen	46.73	50	2.04	48	44.16	13	25.16	17
Yegemen 20	55.52	24	2.61	6	47.19	3	0.73	30
Zhalyn	55.73	22	2.08	45	36.87	54	0.80	2
Zhetisu	56.03	19	2.23	36	39.81	47	0.81	1
Zhadyra	47.02	49	2.09	44	41.33	36	0.75	14
Kazakstanskaya 16	49.07	42	1.55	59	32.55	60	0.72	34
Kazakstanskaya 75	42.17	56	1.81	55	41.86	31	0.68	55
Krasnovodapadskaya	50.71	36	2.08	46	41.71	33	0.71	38
Krasnovodapadskaya 25	48.42	44	2.10	43	42.80	27	0.65	60
Krasnovodapadskaya 210	45.42	54	1.63	58	36.90	53	0.75	15
Karabalykskaya winter	58.02	12	2.42	17	40.60	42	0.74	18
Karabalykskaya ostistaya	47.10	48	1.79	57	38.68	51	0.72	32
Karabalykskaya 101	49.17	41	1.97	50	40.72	41	0.77	8
Konditerskaya	59.77	8	2.20	37	36.22	56	0.76	9
Karasai	57.02	17	2.39	20	41.57	35	0.70	41
Karaspan	36.42	59	2.10	42	40.11	45	0.69	49
Karlygash	51.71	33	2.34	28	44.32	12	0.71	39
Kokbiday	61.73	3	2.51	13	40.22	44	0.68	53
Keremet	60.36	7	2.65	5	43.42	22	0.68	56
Koksu	54.13	26	2.39	21	44.01	15	0.74	23
Kyzylbiday	59.48	9	2.34	27	43.13	26	0.73	25
Knyazhna	52.18	31	2.40	19	41.64	34	0.70	48
Intensivnaya	43.85	55	1.94	53	42.29	29	0.76	10
Manshuk	54.23	25	2.27	34	40.28	43	0.72	35
Myra	53.58	28	2.53	12	46.00	9	0.72	37
Mereke 70	57.48	14	2.54	11	40.05	46	0.73	27
Mereke 75	59.03	10	2.58	9	43.45	21	0.73	26
Matai	53.28	29	2.56	10	45.02	11	0.74	22
Mironovskaya 808	53.73	27	2.36	23	43.30	25	0.69	51
Naz	57.68	13	2.59	8	41.14	38	0.73	28
Nureke	29.40	60	3.06	1	43.34	24	0.74	19
Odesskaya 120	56.02	20	2.35	25	41.81	32	0.75	13
Progress	45.57	53	1.93	54	42.63	28	0.74	20
Prezident	45.82	52	2.19	39	47.82	2	0.72	36
Pyrotrix 50	50.76	35	2.29	31	45.03	10	0.68	54
Taza	49.74	40	2.20	38	44.06	14	0.79	3
Tungysh	46.07	51	1.96	52	43.41	23	0.77	7
Talimi-80	41.40	57	1.81	56	43.71	17	0.69	52
Sultan 2	61.52	4	2.91	2	39.58	48	0.75	12
Sultan 95	48.09	47	2.04	49	43.54	20	0.71	40
Sanzar 8	52.03	32	2.42	16	46.87	4	0.76	11
Sapaly	72.82	1	2.90	3	38.83	50	0.70	46
Steklovidnaya 24	61.37	5	2.26	35	36.33	55	0.74	21
Umanka	49.85	38	2.36	24	46.32	8	0.66	59
Ulugbek 600	49.79	39	2.28	32	35.57	58	0.70	44
Ramin	52.22	30	2.41	18	46.38	7	0.72	33
Raminal	48.37	45	2.06	47	41.99	30	0.69	50
Rasad	58.10	11	2.60	7	43.79	16	0.70	42
Reke	65.10	2	2.78	4	41.18	37	0.73	29
Farabi	56.43	18	2.35	26	40.95	40	0.70	43
Sharora	57.11	15	2.47	15	43.58	19	0.74	24
Yuzhnaya 12	55.68	23	2.31	30	39.01	49	0.78	5
Yubileynaya 60	55.83	21	1.96	51	34.64	59	0.77	6
Yubileynaya 75	48.99	43	2.15	41	43.62	18	0.78	4
Almaly	51.57	34	2.28	33	49.32	1	0.70	47
Bezostaya 1	61.12	6	2.32	29	36.95	52	0.67	57
Bayandy	50.17	37	2.37	22	46.71	6	0.74	16
Bogornaya 56	57.10	16	2.48	14	46.80	5	0.73	31
2017/2018	50.22	3	2.22	3	42.01	1	0.74	1
2018/2019	55.63	1	2.31	1	41.67	3	0.71	2
2019/2020	51.36	2	2.27	2	41.91	2	0.71	3
Total average	52.40		2.27		41.86		0.72	

GN-grain number per spike (pieces); GW-grain weight per spike (grams); TKW-thousand kernel weight (grams); NDVI-Normalized difference vegetation index (unit).

Among 60 studied Kazakh and foreign wheat varieties the *Bt8* gene was detected in 12, the *Bt10* gene in 6, the *Bt11* in 7, and two *Bt8* as well as *Bt11* genes in 2 varieties. The *Bt9* gene was described in 15 varieties. In the Karasai cultivar, a combination of four resistance genes, such as *Bt8, Bt11, Bt9*, and *Bt10* were identified. Three genes (*Bt8, Bt9, Bt10*) were found in the varieties of Dinara, Egemen 20, and 3 genes (*Bt8, Bt9, Bt11*) were found in the following varieties Kazakstanskaya 75 and Naz. The varieties such as Zhetisu, Mereke 75, Sultan 2, Sultan 95, Sapaly were sources of two *Bt* genes *Bt9, Bt11* and *Bt8, Bt9,* respectively.

To identify donors and sources of resistance to *T. caries*, molecular screening and phytopathological evaluation of 60 wheat varieties were performed. Among 60 varieties of wheat from 2018 and 2019, 17 showed a highly resistant reaction, with a 0% level. These varieties include Dinara, Yegemen 20, Zhalyn, Karasai, Kyzylbiday, Matai, Progress, Taza, Talimi-80, Sultan 2, Sapaly, Steklovidnaya 24, Raminal, Farabi, Yuzhnaya12, Yubileynaya 75 and Almaly. Because of phytopathological evaluation of 60 Kazakh wheat varieties on an artificial infectious condition during 3 years were determined 32 genotypes, resistant to common bunt.

## 4 Discussion

The results of this analysis confirm the effectiveness of the identified *Bt* genes such as Bt8, Bt9, Bt10, and Bt11 against races of *T. caries* on wheat in the Sothern-east region of Kazakhstan according to what many other scientists have accented to in previous studies around the world (Blazkova and Bartos, 2002, Liatucas and Ruzgas, 2018. The resistance genes *Bt8, Bt9* and *Bt10* derived from PI178383 are already integrated in several wheat varieties. These collections produce resistant to prevalent races of *T. caries* and T*. laevis* and are use as parents for the development of resistant varieties to common bunt. The resistance genes *Bt8, Bt9* and *Bt10* were effective in Austria (Hagenguth, 2016). Previous studies referred the effectiveness of the resistance genes *Bt1, Bt3, Bt9, Bt11* and *Bt12* in Iraq (Al-Maaroof et al., 2016) and resistance genes *Bt5, Bt8, Bt9, Bt10* and *Bt11* in Syria (Mamluk and Nachit, 1994) as well as resistance genes Bt6, Bt9, Bt11, Bt12, Bt13, Bt15, and Btp in Nebraska, USA (Mourad et al., 2018).

As a result of our previous researches on common bunt resistance of advanced winter wheat varieties, we suggested 23 sources that are resistant to the disease. Molecular screening of wheat varieties to common bunt showed that the resistance genes Bt10 and Bt9 identified in 17 (Kazakh, Hungarian and Bulgarian) winter wheat varieties (Madenova et al., 2019a, Madenova et al., 2019b, Madenova et al., 2020).

The results of our previous and current research confirm the effectiveness of the known resistance genes *Bt2, Bt3, Bt4, Bt5, Bt6, Bt8, Bt9, Bt10, Bt11, Bt12, Bt13, Bt14, Bt8, Bt9 and Bt10* to the common pathogens *T. caries* and *T. laevis* with what has been mentioned by many other researchers in previous studies in the world (Al-Maaroof et al., 2016, Koishybayev, 2018). The presence of these Bt-genes in some wheat varieties gives the opportunity to encourage their implementation in the common bunt breeding program as parents for crossing with high yield susceptible varieties. The limited knowledge regarding the genetic basis of bunt resistance impedes the application of MAS for the rapid development and adoption of bunt resistant varieties, which are needed for organic and low input agricultural production of wheat. In this study, the common bunt resistance genes such as *Bt8, Bt9, Bt10* and *Bt11* from Kazakh and foreign wheat varieties to were described. These genes confer resistance to most Kazakh common bunt isolates and races, making it a favorable target for introgression into elite breeding materials.

## 5 Conclusion

In summary, this investigation summarized that molecular markers are a convenient and efficient approach to identify effective common bunt resistance genes in wheat varieties, and particularly so where a well-characterized pathogen collection is not available for multi-pathotype assessments. In conclusion, out of 60 Kazakh and foreign wheat genotypes, 3 varieties were found with the *Bt8, Bt9,* and *Bt10* (Dinara, Yegemen 20, Karasai) as well as 3 varieties with the Bt8, Bt9, and Bt11 (Kazakhstanskaya 75, Karasai and Naz). Carriers of the *Bt10* gene was found in the 6 entries such as Dinara, Yegemen 20, Karabalykskaya ostistaya, Karasai, Kokbiday, and Almaty. The resistance genes such as *Bt8, Bt10, Bt11, Bt9,* and *Bt10* were identified in the winter wheat Karasai mixture. The sources of the *Bt8* gene was found in the 10 varieties and *Bt9* in 15 varieties. The *Bt11* gene identified in 7 varieties. Results of phytopathological and molecular screening of Kazakh and foreign wheat genotypes shown that 12 varieties with resistance genes to the disease, which were resistant (R) to artificial infection. Marker-assisted selection can be efficiently applied to develop wheat cultivars with effective gene combinations that would directly assist in developing durable resistance in Kazakhstan. Among the studied wheat varieties, we selected 22 entries, which are sources of resistance genes to the disease according to estimates of the artificial infectious conditions. These varieties represented an immune type of reaction to the disease. We recommend using these sources as donors in future breeding programs.

## Contribution to this manuscript

All authors contributed equally.

## Funding

The National Grant Program of Kazakhstan financially supported this work for 2018–2020. The Ministry of Education of the Republic of Kazakhstan provided funding within the framework of the budget programme 055 ‘Scientific and/or technical activities’ and subprogram 102 ‘Grant funding of scientific research’, contract No. 176 of 15 March 2018, No. AP05131091.

## Declaration of Competing Interest

The authors declare that they have no known competing financial interests or personal relationships that could have appeared to influence the work reported in this paper.
